# Vectors, host range, and spatial distribution of *Dirofilaria immitis* and *D. repens* in Europe: a systematic review

**DOI:** 10.1186/s40249-025-01328-2

**Published:** 2025-07-02

**Authors:** Carolin Hattendorf, Renke Lühken

**Affiliations:** Bernhard Nocht Institute for Tropical Medicine, Hamburg, Germany

**Keywords:** *Dirofilaria immitis*, *Dirofilaria repens*, Spread, Globalisation, Climate warming, Europe

## Abstract

**Background:**

*Dirofilaria immitis* and *D. repens* are mosquito-borne nematodes with dogs as primary hosts, but other mammalian species including humans can be also infected. In the last century, circulation of both pathogens was predominantly restricted to Southern Europe. However, different studies indicated a potential establishment in Central, Eastern and Western parts of Europe as an increasing threat to animal and human health. Therefore, we conducted a systematic literature review of *Dirofilaria* data in Europe to give a comprehensive overview of potential mosquito vectors and vertebrate hosts, including the collection of different metadata (e.g. sampling year and site), allowing to analyse the spread pattern of the parasites in Europe.

**Methods:**

On 24 January 2022, we conducted a systematic literature review of all available publications in the PubMed database reporting *D. immitis* and *D. repens* screening in mosquitoes and mammalian vertebrates in Europe. We only included acute infection of *Dirofilaria* spp., i.e. excluding studies only screening antibodies, and in addition noted the travel history and the accuracy of the sampling locations. These data were used to analyse the range of potential vectors and hosts and for a comparison of the spatial distribution between the twentieth and twenty-first century.

**Results:**

Both nematodes appear to have a high overlap of *Aedes*, *Anopheles* and *Culex as* potential vector species, which are abundant in Europe. Most published *D. immitis* infections were reported in dogs, while *D. repens* predominantly were reported in humans. *Dirofilaria immitis* infections were detected in a wider range of wild and zoo animals. Compared to the last century, many more countries especially in Central Europe were affected by *Dirofilaria* spp. circulation, illustrating a significant spread over the last 20 years.

**Conclusions:**

Our findings suggest that *D. immitis* and *D. repens* are a growing health concern for animals and humans in Europe. Continuous globalisation and climate warming will probably lead to a further spread and increased circulation in the future. All data are made available open access, which will enable further analysis.

**Graphical Abstract:**

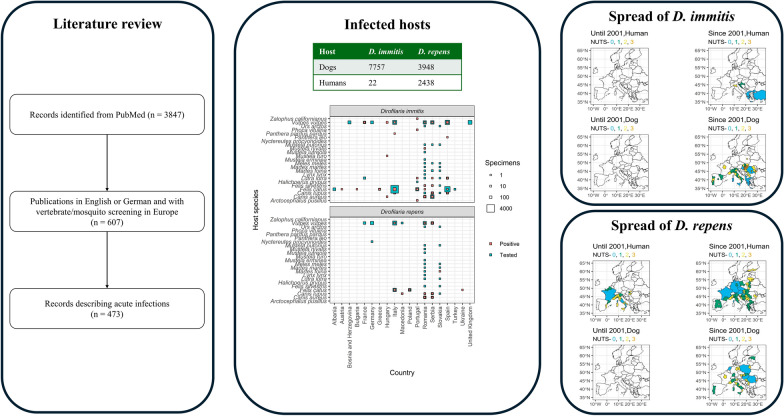

**Supplementary Information:**

The online version contains supplementary material available at 10.1186/s40249-025-01328-2.

## Background

Two *Dirofilaria* species are present in Europe: *D. immitis* (Leidy, 1856) and *D. repens* (Railliet & Henry, 1911) [1]. Both circulate in an enzootic cycle between mosquitoes and domestic dogs (*Canis familiaris*), although other carnivores like red foxes (*Vulpes vulpes*) and grey wolves (*Canis lupus*) can also be infected (e.g. *Dirofilaria*-positive tested grey wolves in Italy and Spain [2, 3] or red foxes in France and Germany [4, 5]). Mosquitoes are infected with microfilaria during blood-feeding on an infected host, which then develop to infective larvae in susceptible vectors [6]. *Dirofilaria* can be transmitted to other mammals, such as humans (*Homo sapiens*) and rodents (Rodentia), although these are generally ‘dead-end’ hosts [6], i.e. no development of microfilaria occurs. *Dirofilaria immitis* localise in the pulmonary arteries of dogs, where they sexually reproduce and release microfilariae into the bloodstream [1, 7]. Infections can lead to severe disease in dogs and cats (*Felis catus*) with symptoms ranging from chronic cough to heart failure [8, 9]. In humans, *D. immitis* mostly forms pulmonary nodes, which are generally asymptomatic, but frequently mistaken with lung cancer in radiography [6]. However, some humans develop severe symptoms including fever, chest pain, coughing, haemoptysis, wheezing arthralgia or malaise [10]. *Dirofilaria repens* generally localises subcutaneously [1, 6]. Approximately 35% of human *D. repens* infection cases occur in the ocular region, which can lead to impaired or a complete loss of vision [11]. Around 10% of affected patients suffer permanent complications like retinal detachment or glaucoma [12]. Notably, there have been a few reported cases where viable *D. repens* microfilariae have been found in the blood stream of infected humans [13–17], but these seem to be rare exceptions. The majority of human *Dirofilaria* infections in Europe are caused by *D. repens* [18], while the majority of reported *Dirofilaria* cases in dogs are *D. immitis* [1]. However, it has to be noted that *D. immitis* is easier to diagnose in dogs because it more often leads to severe symptoms in dogs and respective tests are available [19].

First cases of human dirofilariasis presumably were diagnosed in 1566 in a Portuguese girl [20] and 1626 in an Italian dog [21] for *D. repens* and *D. immitis*, respectively. In the twentieth century, autochthonous circulation of these parasites was predominantly reported from the Southern parts of Europe, but currently there are increasing reports of a spread towards Central, West and East Europe [22]. Many previously *Dirofilaria*-free countries are now considered endemic [23]. Climate warming is thought to be the main reason, allowing the successful development of the nematodes in the mosquito [24–26]. Another important factor is the movement of dogs in Europe, which was made considerably easier with European regulations for traveling with pets [27]. To gain a better picture of the potential vector range and spatial expansion of *D. immitis* and *D. repens* in Europe over the last two centuries, we conducted a systematic literature review of *Dirofilaria* data in mosquitoes and vertebrate hosts, including the collection of different metadata (e.g. sampling year and site).

## Methods

All published articles matching the keyword ‘dirofilaria’ in any search field recorded in PubMed [28] were extracted on 24 January 2022, which can serve as a starting point for future updates. Papers were selected using the following inclusion criteria: (1) article language English or German, (2) a host was diagnosed with an acute infection of *Dirofilaria* spp., i.e. excluding studies only screening antibodies, and (3) the sampling was conducted in Europe. The following information was extracted from each publication: country, date of diagnosis/sampling, sampling location, host species, travel history, screening method, number of tested and number of positive specimens per *Dirofilaria* species. In addition, for mosquito studies the mosquito trap and pooling information (pool size, body part, etc.) were noted.

If the date of diagnosis was not specified, the date of publication was used and if only a sampling period was given, the total number of cases was split evenly across the sampling years. The accuracy of the sampling locations was classified to decide which level of the Nomenclature of Territorial Units for Statistics (NUTS) classification of the European Union [29] was used for visualisation of parasite distribution in humans, dogs and other vertebrate hosts: ‘very high’ (coordinates or address, NUTS-3 level), ‘high’ (town or specific area, NUTS-2 level), ‘medium’ (hospital or greater area (e.g. county), NUTS-1 level), and ‘low’ (country, NUTS-0 level). For the spatial analysis of the *Dirofilaria* distribution, we only included reports with unremarkable travel history. However, many studies did not include any information on the travel history. Therefore, we also conducted the spatial visualisation with all unremarkable and unknown travel history for the supplement. Reports with a known travel history were excluded from analysis. Furthermore, we compiled visual summaries of country-specific *Dirofilaria* screening results from mosquitoes and less common vertebrate hosts, excluding humans and dogs. All computational analysis was performed in R (Version: 4.2.2) using the R-Studio IDE (Version:2022.12.0) [30]. Additionally, functions from the following packages were used for data preparation, visualization and analysis: terra [31], tidyterra [32], geodata [33], readxl [34], ggpubr [35], plyr [36], dplyr [37], and ggplot2 [38].

## Results and discussion

### Characteristics of published studies

**Fig. 1:**
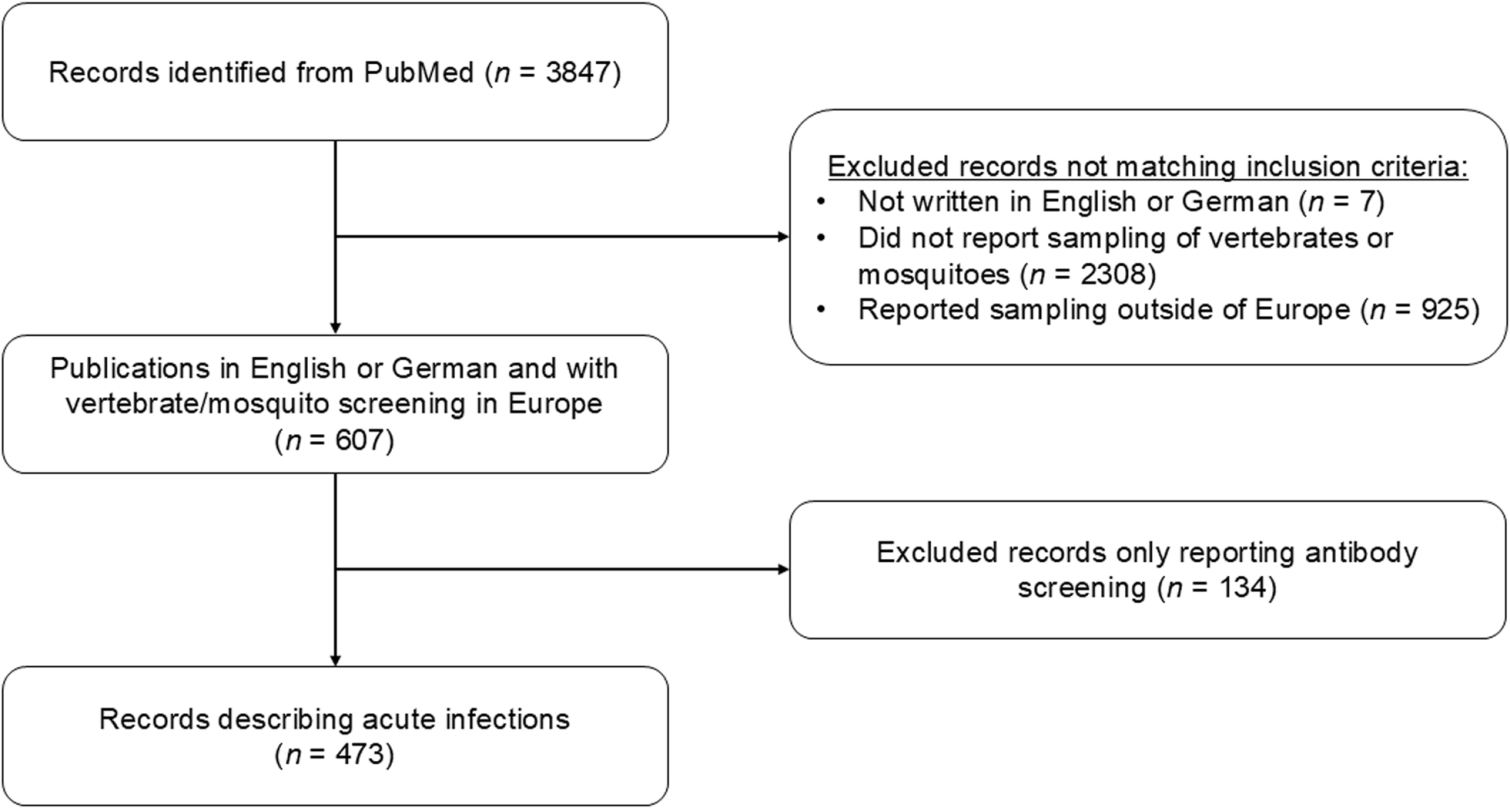
Flow diagram presenting the search process, including inclusion and exclusion criteria for articles screen

**Fig. 2:**
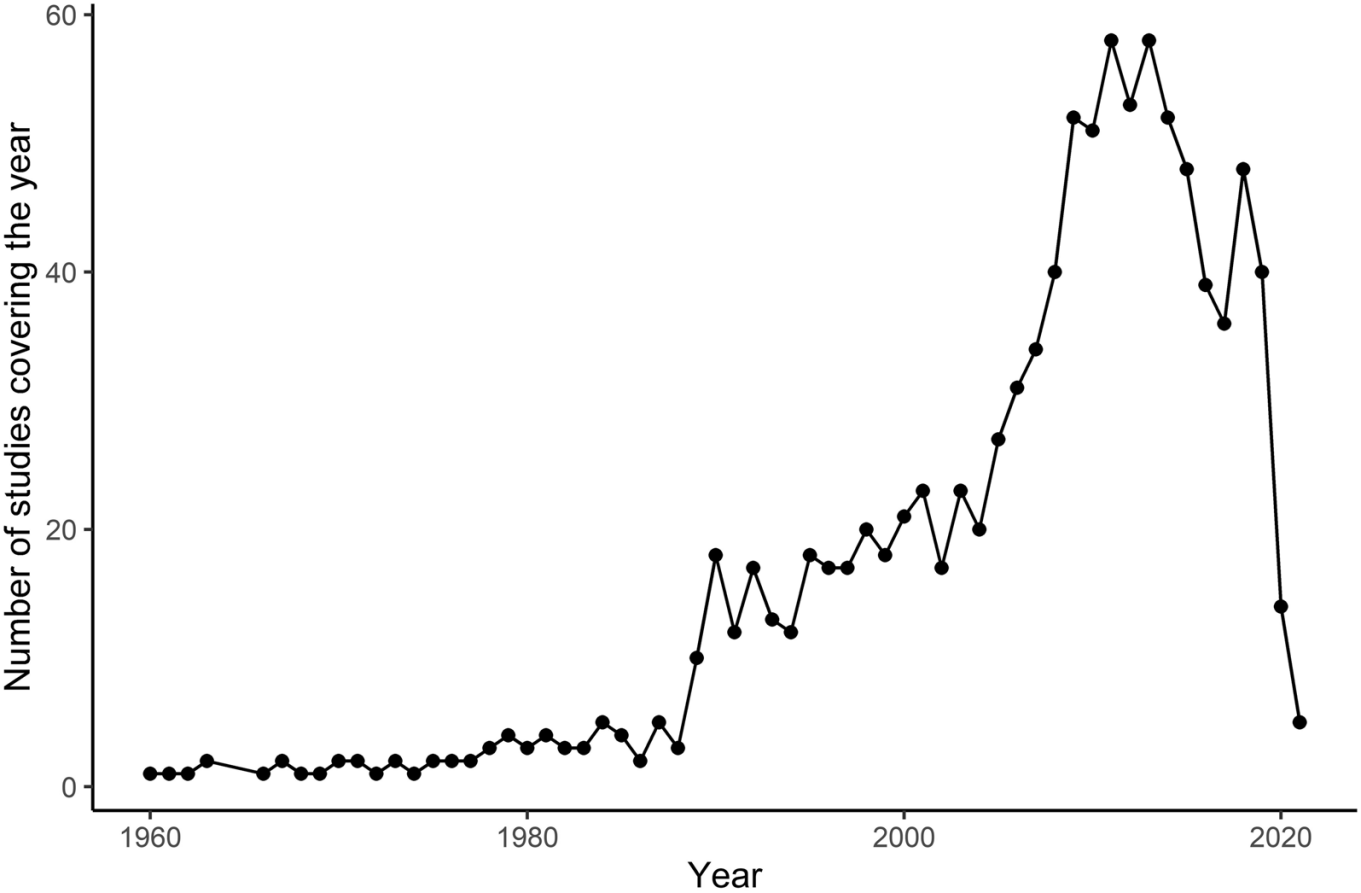
Number of studies reporting *Dirofilaria immitis* and *D. repens* in Europe

A total of 3847 publications were extracted from PubMed. Of these, 473 (12.3%) matched our inclusion criteria (Fig. 1). We observed an increase in publications reporting *Dirofilaria* from the beginning of the 1990s and another increase in the mid-2000s (Fig. 2).

The number of publications reporting *Dirofilaria* spp. infections have increased in the last two decades compared to the previous century [18, 22]. This is most likely driven by both increased research and awareness, but also the spread of the parasites [25, 39]. *Dirofilaria immitis* and *D. repens* have to be considered endemic in countries that were considered to be *Dirofilaria*-free in the twentieth century, e.g. Czech Republic [40, 41].

### *Dirofilaria* infections of mosquitoes

**Fig. 3:**
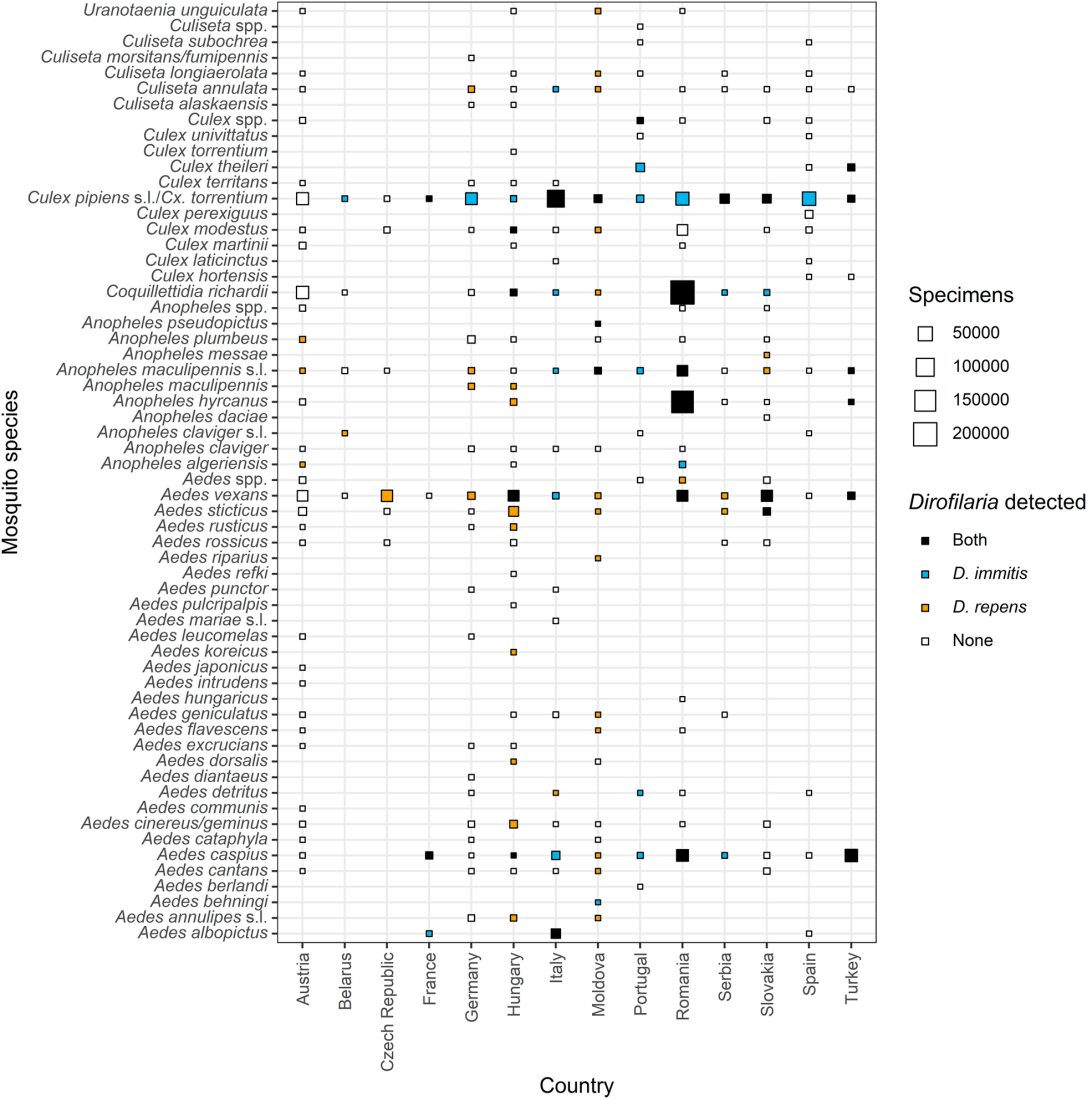
*Dirofilaria immitis* and *D. repens* reports in mosquitoes for different European countries

Thirty-eight publications (8.0%) included screenings of mosquitoes for *Dirofilaria* with a total of 1,658,041 specimens tested over 62 mosquito taxa collected in 14 different European countries (Fig. 3). *Dirofilaria immitis* was detected in 17 different mosquito taxa from 12 countries, most frequently in *Culex pipiens* s.l. (11 countries) and *Aedes caspius* (7 countries). *Dirofilaria repens* infections were reported for 31 different mosquito species from 13 countries with *Aedes vexans* (8 countries), *Cx. pipiens* s.l. (6 countries) and *Anopheles maculipennis* s.l. (6 countries) most frequently found positive. A total of 15 mosquito taxa were found positive for both *Dirofilaria* species. *Dirofilaria immitis* was exclusively detected in *Ae. behningi*, while *D. repens* was exclusively found in 16 different taxa of the *Aedes*, *Anopheles*, *Culiseta* and the *Uranotaenia* genus, e.g. *Ae. cantans*, *An. claviger*, *Cs. annulata* or *Ur. unguiculata*. Most studies on *Dirofilaria* prevalence in mosquitoes focused on Southern and Eastern Europe, but some studies also confirmed autochthonous circulation in Central Europe, e.g. Austria or Germany.

There is a huge overlap between the potential vector species for *D. immitis* and *D. repens*, which are widespread in Europe and show host-feeding patterns with a substantial proportion of mammals, e.g. *Cx. pipiens* s.l. or *An. maculipennis* s.l. [42–44]. Interestingly, the exotic *Ae. albopictus* was much more often reported to be infected with *D. immitis* than *D. repens*. This mosquito species has been implicated as an important driver of the spread of *Dirofilaria* [22, 26, 45].

### *Dirofilaria* infections of animals

**Fig. 4:**
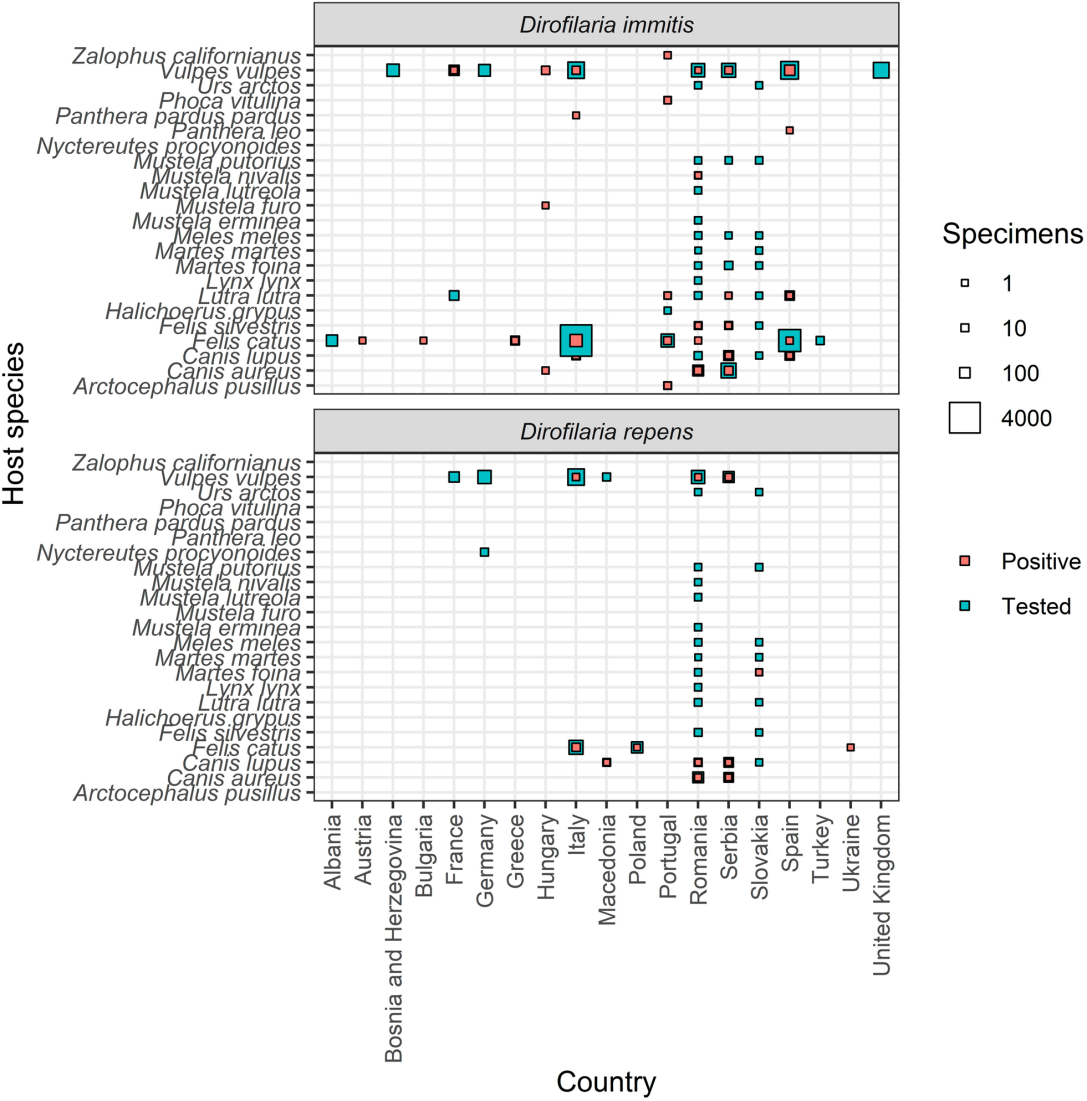
*Dirofilaria immitis* and *D. repens* reports in vertebrates except humans and dogs for different European countries

A total of 198 publications (accounting for 41.9% of the included publications) reported dog infections, with a total of 11,713 cases. Of these, 7757 (66.2%) were identified as *D. immitis*, 3948 (33.7%) as *D. repens*, and eight (0.1%) were not further differentiated *Dirofilaria* species. In 199 publications (42.1%), human *Dirofilaria* spp. infections were described, summing up to 2555 reported human cases, of which the majority of 2438 (95.4%) were *D. repens*, followed by 95 (3.7%) not further specified *Dirofilaria* spp. and 22 (0.9%) *D. immitis*. Only 33 publications (7.0%) reported *Dirofilaria* infections in cats (278 cases): 252 (90.1%) *D. immitis*, 24 (8.6%) *D. repens* and two (0.7%) not further specified *Dirofilaria* species. In addition, 59 publications (12.5%) described *Dirofilaria* infection in other mammals, the majority of which were caused by *D. immitis* (Fig. 4). These studies predominantly focused on domestic cats (34 publications, 7.2%) and red foxes (15 publications, 3.2%). In addition, *Dirofilaria* were detected in a wide variety of wild carnivores [e.g. golden jackal (*Canis aureus*), grey wolf or Eurasian otter (*Lutra lutra*)] and zoo animals [e.g. lion (*Panthera leo*) or California sea lion (*Zalophus californianus*)]. A wider variety of vertebrate hosts was studied in Slovakia, Serbia and Romania, while studies in other countries focused on few potentially infected species like red foxes or only reported single cases.

Most infections were reported from dogs as the primary host of *Dirofilaria* [1]. The majority of these cases were caused by *D. immitis*, which is well known to cause a more severe disease in dogs compared to *D. repens*, leading to a higher probability of diagnosis [7]. Additionally, rapid tests are only available for *D. immitis* and not *D. repens.* Therefore, *D. repens* might be underreported and its actual prevalence among dogs is probably higher [11]. Besides dogs and humans, there were also several reports of infections in cats, although it is assumed that cats do not play an important role for *Dirofilaria* transmission [46]. Similarly, several other mammalian species diagnosed with an infection were held in zoos or as pets, allowing diagnosis [47–50]. Furthermore, there are some wild animals in which *Dirofilaria* infections were identified, predominantly in canids like red foxes [4, 5, 51–62], golden jackals [52–55, 63, 64], and grey wolves [2, 3, 51, 52, 55, 65, 66]. Zoo and wild animals were almost always infected by *D. immitis*, which again might be because *D. immitis* in comparison to *D. repen*s infections more often leads to severe symptoms, corresponding test kits are available or because *D. immitis* has a broader host range. However, it has to be kept in mind that the higher number of publications on wild or uncommon hosts compared to dogs probably are attributed to a publication bias favouring novel finding in wildlife species. Research on dogs in endemic countries might be perceived as less interesting, while studies on bears, wolves, and foxes are often considered more scientifically interesting.

### *Dirofilaria* infections of humans

**Fig. 5:**
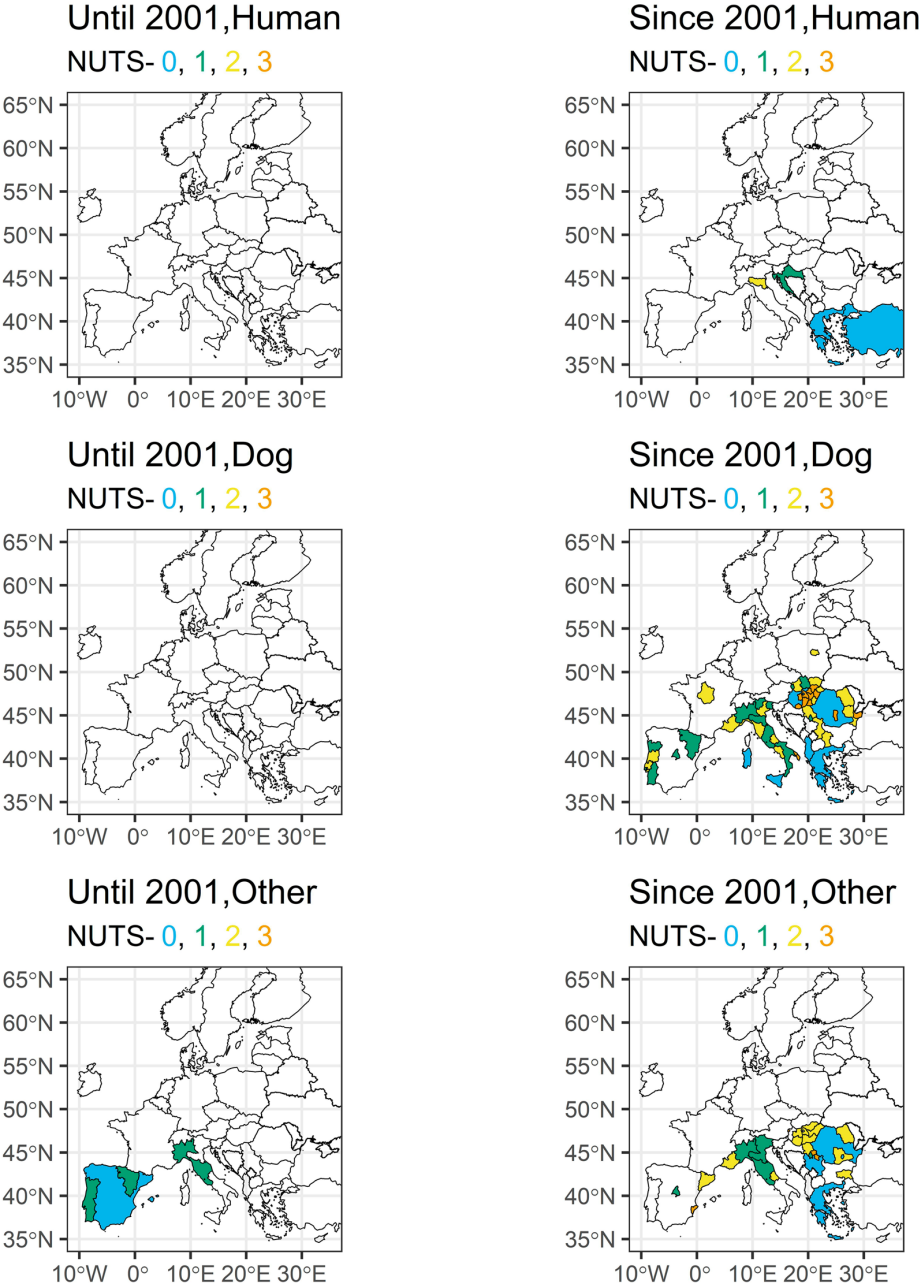
*Dirofilaria immitis* cases in humans, dogs and other mammals with unremarkable travel history in Europe until and since 2001 at different geographical levels

**Fig. 6:**
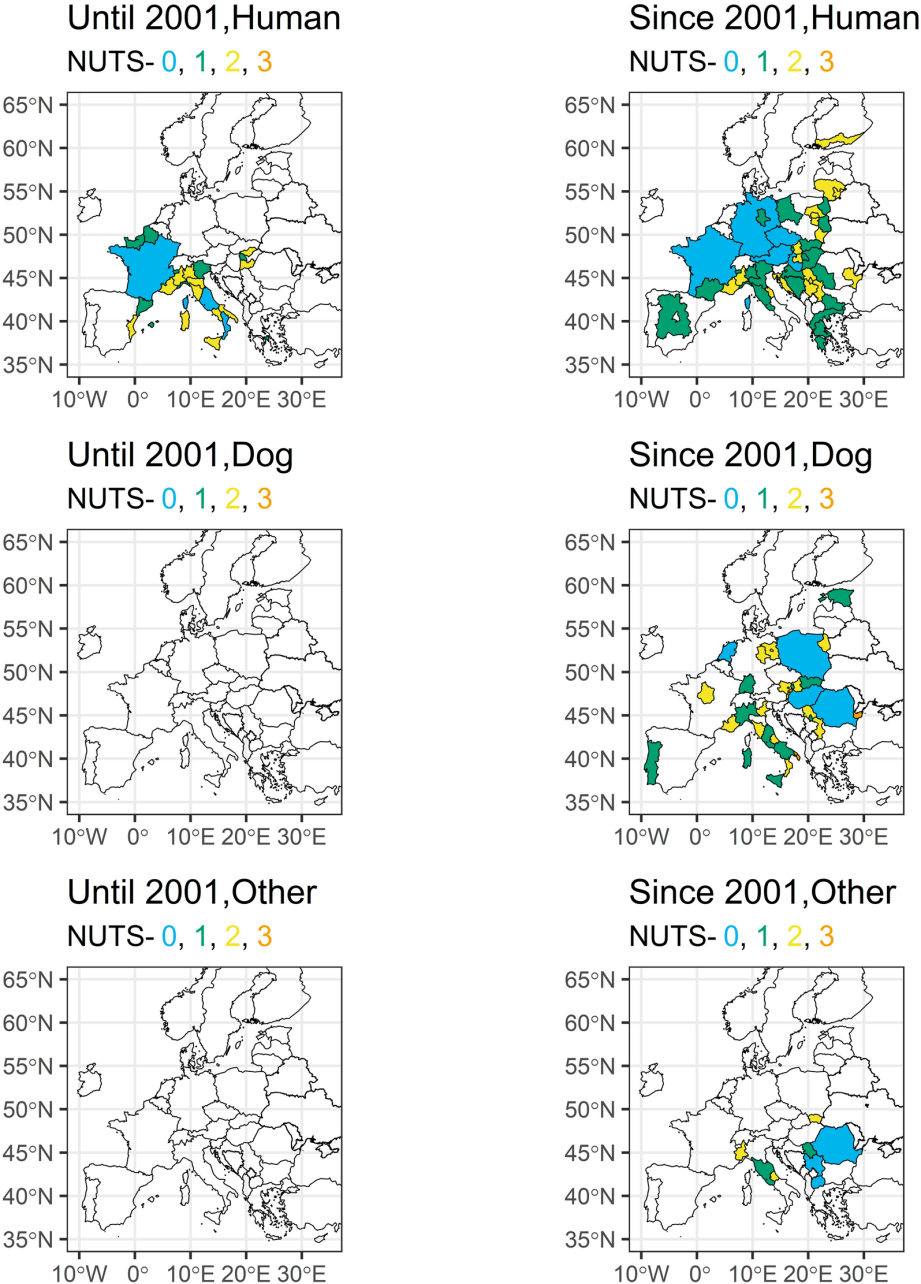
*Dirofilaria repens* cases in humans, dogs and other mammals with unremarkable travel history in Europe until and since 2001 at different geographical levels

Only focusing on the studies with unremarkable travel history, the majority of the few *D. immitis* cases in dogs and other mammals until 2001 were recorded in Southern Europe, particularly in Spain, Italy and Portugal (Fig. 5; see supplementary file 1 and supplementary file 2 for visualisation of all cases with unremarkable and unknown travel history). No human cases were reported before 2001. In the twenty-first century, *D. immitis* infections were found in most countries of South and Central Europe and even in Central Europe, such as Poland and France. A wide distribution in particular was confirmed in dogs and other mammals for wide parts of Eastern Europe and Italy. *Dirofilaria repens* infections, especially looking into human cases, were reported much more widespread than *D. immitis* already during the twentieth century in particular for various regions in Italy and France, while dogs were only tested positive in Italy and Spain (Fig. 6). We observed a strong increase of affected countries for both humans and dogs, including countries in Eastern and Southern Europe (e.g. Ukraine, Slovakia, Greece), but also Central Europe including the Netherlands, Germany or Poland. The most Northern infection was reported in humans from Finland.

In contrast to dogs, the overwhelming majority of cases in humans were caused by *D. repens*, confirming previous observations that most human *Dirofilaria* infections in Europe are caused by this species [67, 68]. The reason for this remains unclear, as human infections with *D. immitis* are possible in general and reported frequently [69]. One hypothesis suggested that European *D. immitis* might be genetically distinct from *D. immitis* found in other regions, making it less capable of surviving within humans [11]. However, this hypothesis has later been disproven [70, 71]. Another explanation could be that *D. repens* influences the circulation of *D. immitis*, e.g. it has been shown for Southern Italy that *D. repens* impedes the spread of *D. immitis* in dogs [72]. If this plays a general epidemiological role and if this is also true for humans requires further research. Furthermore, it has been proposed that *D. repens* is more difficult to control, because, as mentioned above, rapid tests are only available for *D. immitis* and current preventative and curative treatments are designed for *D. immitis* and are not as effective against *D. repens* [18]. Additionally, *D. repens* infections are often asymptomatic in dogs which might lead to a longer time period where a dog is infective, and a mosquito can ingest and transmit the parasite to further hosts [6, 73].

### Spread of *Dirofilaria* in Europe

It is undeniable that both, *D. immitis* and *D. repens*, are spreading in Europe and more humans and animals are at risk of infection. In part this might be also a diagnostic artefact, i.e. imported and travelling dogs are more routinely tested, which leads to more detection [39]. Transport of pets has significantly increased during the twenty-first century as a consequence of the Pet Travel Scheme, which was introduced by the European Union in 2000 and made travel of companion animals significantly easier and led to an increase in imported cases [27]. Another reason is the continuously high number of stray dogs in some countries, which are not subject to regular treatment and act as reservoirs for the parasites, e.g. countries with many stray dogs, such as Romania or Bulgaria continue to regularly report *Dirofilaria* spp. cases [74–76]. Besides climate warming and stray dogs, outdoor keeping of dogs is considered a crucial factor, leading to a higher risk for mosquito bites compared to dogs kept inside [45]. However, probably one of the most important factors for the spread of *Dirofilaria* is climate warming. Higher temperatures lead to faster development of *Dirofilaria* larvae inside the mosquito vector [77, 78]. Prolonged warm periods extend the transmission season [11]. There is a significant increase in areas at risk, especially in more Northern countries. This spread has been predicted since the early 2000s [25, 73] and with continuous climate warming will further increase in the future. Finally, increasing temperatures in Europe also allowed the widespread establishment of exotic, daybiting mosquito species such as *Ae. albopictus* [79–81], which is an important potential vector for *D. immitis* and *D. repens* and the establishment of the species in numerous European regions has been linked to increased *Dirofilaria* circulation [22]*.*

### Limitations of this study

There are several limitations of this literature study. As discussed in detail above, there is a significant difference in the severity of disease in the animal host between *D. repens* and *D. immitis*. Therefore, *D. repens* infections are probably underreported, particularly in dogs, due to the lack of rapid diagnostic tests. At the same time, more research is needed to understand the factors that make *D. repens* more successful in establishing infections in humans compared to *D. immitis*. In general, while we discuss the role of climate change and pet movements in the spread of *Dirofilaria*, other potential factors that may influence the spatial distribution and expansion of these parasites should be explored in the future, e.g. landscape changes. Finally, the studies on *Dirofilaria* in mosquitoes and vertebrates (Figs. 3, 4) indicate a spatial bias toward countries in the Mediterranean and Southeast Europe, while additional studies in Central and Northern Europe are necessary to accurately assess the local risk of *Dirofilaria* transmission.

## Conclusions

*Dirofilaria immitis* and *D. repens* are an increasing threat to veterinary and public health in Europe. Both parasites have dramatically expanded their circulation area and are now endemic in areas that were considered *Dirofilaria-*free only one or two decades ago [23]. The warming climate and the abundant presence of potential competent vectors allows the establishment of the parasites in Central Europe, e.g. Germany and Poland. Due to their rising relevance in animal and human health, a Europe-wide unified surveillance system similar to the system in the United States [82] should be implemented in order to better understand the change of circulation patterns and to plan and execute preventative strategies, e.g. dog treatment. All data and code are provided as open access, allowing for future analyses (https://github.com/luehkenecology/dirofilaria_review_europe).

## Supplementary Information

Supplementary material 1. *Dirofilaria immitis* cases in humans, dogs and other mammals with unremarkable and unknown travel history in Europe until and since 2001 at different geographical levels.

Supplementary material 2. *Dirofilaria repens *cases in humans, dogs and other mammals with unremarkable and unknown travel history in Europe until and since 2001 at different geographical levels.

## Data Availability

The datasets supporting the conclusions of this article and all codes used for data analysis are available at https://github.com/luehkenecology/dirofilaria_review_europe

## Abbreviation

NUTS: Nomenclature of Territorial Units for Statistics
