# Supplementary figures and images for: Vectors, host range, and spatial distribution of Dirofilaria immitis and D. repens in Europe: a systematic review
Source: Infect Dis Poverty. 2025 Jul 2;14:58. doi: 10.1186/s40249-025-01328-2 (PMC12217203; doi:10.1186/s40249-025-01328-2)

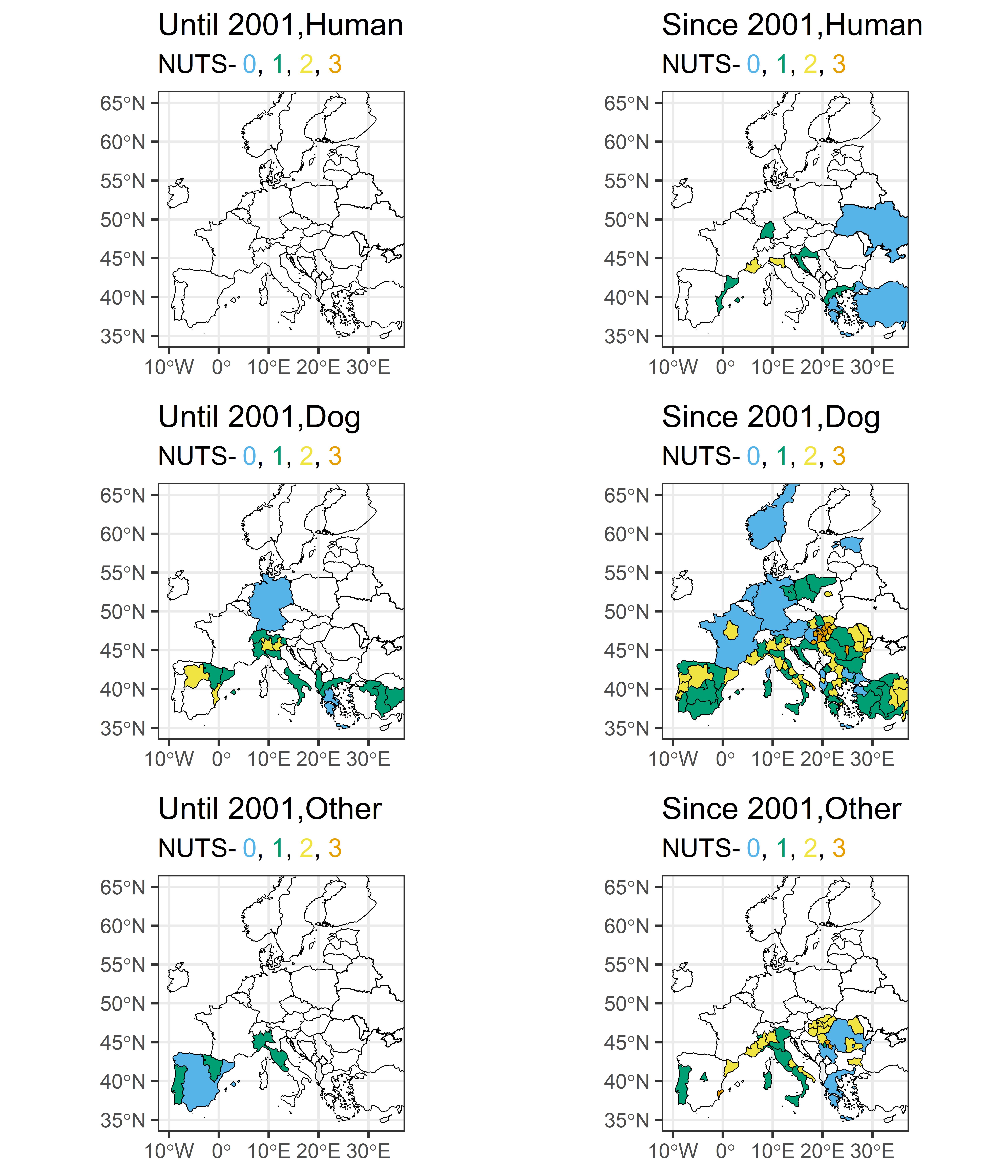

Supplement: Supplementary file 1 — Supplementary material 1. Dirofilaria immitis cases in humans, dogs and other mammals with unremarkable and unknown travel history in Europe until and since 2001 at different geographical levels. [file 40249_2025_1328_MOESM1_ESM.jpg]

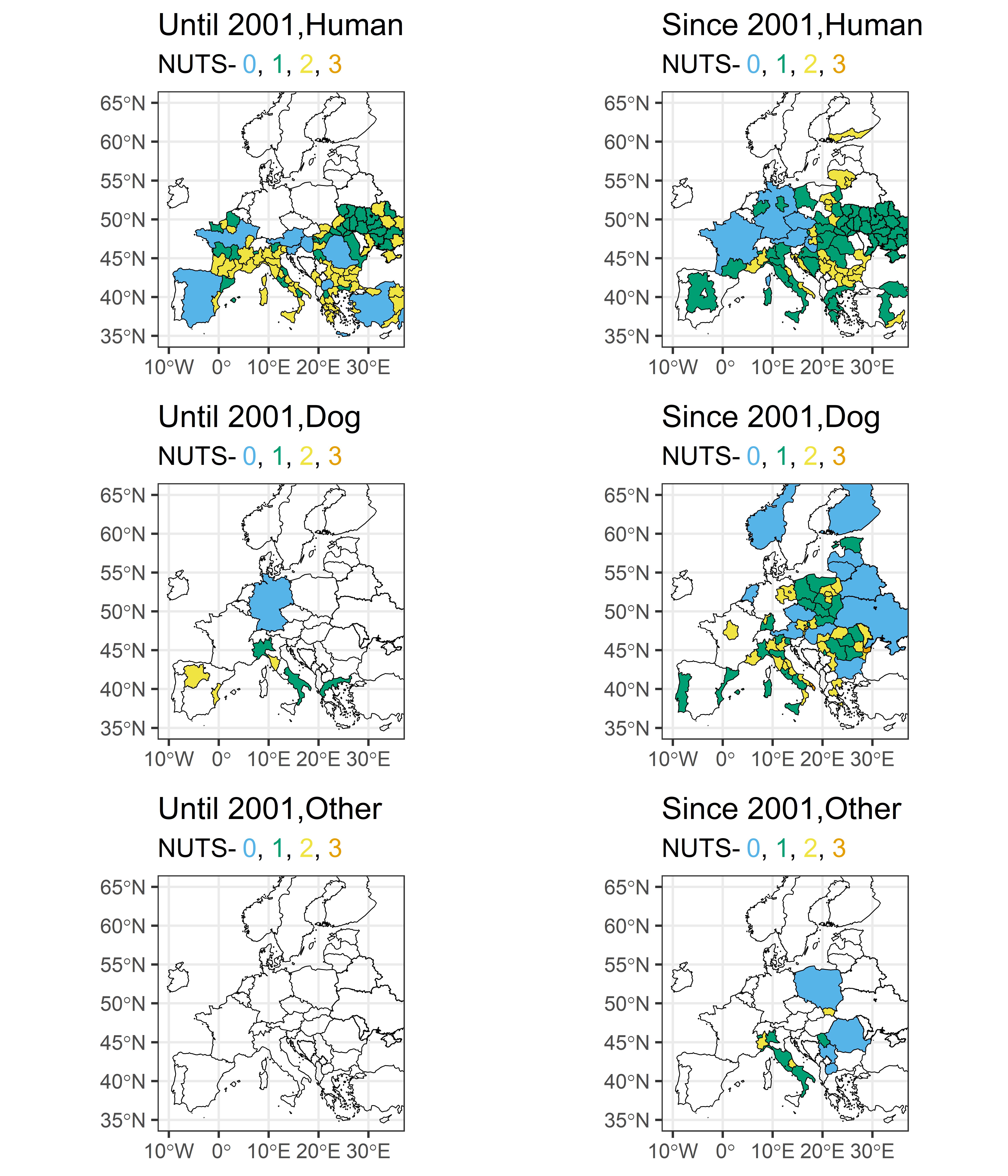

Supplement: Supplementary file 2 — Supplementary material 2. Dirofilaria repens cases in humans, dogs and other mammals with unremarkable and unknown travel history in Europe until and since 2001 at different geographical levels. [file 40249_2025_1328_MOESM2_ESM.jpg]
